# Growth and Photosynthetic Responses of Cowpea Genotypes under Waterlogging at the Reproductive Stage

**DOI:** 10.3390/plants11172315

**Published:** 2022-09-04

**Authors:** Omolayo J. Olorunwa, Bikash Adhikari, Skyler Brazel, Ainong Shi, Sorina C. Popescu, George V. Popescu, T. Casey Barickman

**Affiliations:** 1 North Mississippi Research and Extension Center, Department of Plant and Soil Sciences, Mississippi State University, Verona, MS 38879, USA; 2 Department of Horticulture, PTSC 316, University of Arkansas, Fayetteville, AR 72701, USA; 3 Department of Biochemistry, Molecular Biology, Entomology, and Plant Pathology, Mississippi State University, Mississippi State, MS 39762, USA; 4 Institute for Genomics, Biocomputing, and Biotechnology, Mississippi State University, Mississippi State, MS 39762, USA

**Keywords:** *Vigna unguiculata*, biomass, stomatal conductance, photochemistry, leaf gas exchange, hypoxia, recovery, waterlogging tolerance

## Abstract

Waterlogging is an important environmental stress limiting the productivity of crops worldwide. Cowpea (*Vigna unguiculata* L.) is particularly sensitive to waterlogging stress during the reproductive stage, with a consequent decline in pod formation and yield. However, little is known about the critical processes underlying cowpea’s responses to waterlogging during the reproductive stage. Thus, we investigated the key parameters influencing carbon fixation, including stomatal conductance (g_s_), intercellular CO_2_ concentration, chlorophyll content, and chlorophyll fluorescence, of two cowpea genotypes with contrasting waterlogging tolerance. These closely related genotypes have starkly contrasting responses to waterlogging during and after 7 days of waterlogging stress (DOW). In the intolerant genotype (‘EpicSelect.4’), waterlogging resulted in a gradual loss of pigment and decreased photosynthetic capacity as a consequent decline in shoot biomass. On the other hand, the waterlogging-tolerant genotype (‘UCR 369’) maintained CO_2_ assimilation rate (*A*), stomatal conductance (g_s_), biomass, and chlorophyll content until 5 DOW. Moreover, there was a highly specific downregulation of the mesophyll conductance (g_m_), maximum rate of Rubisco (V_cmax_), and photosynthetic electron transport rate (J_max_) as non-stomatal limiting factors decreasing *A* in EpicSelect.4. Exposure of EpicSelect.4 to 2 DOW resulted in the loss of PSII photochemistry by downregulating the PSII quantum yield (F_v_/F_m_), photochemical efficiency (Φ_PSII_), and photochemical quenching (qP). In contrast, we found no substantial change in the photosynthesis and chlorophyll fluorescence of UCR 369 in the first 5 DOW. Instead, UCR 369 maintained biomass accumulation, chlorophyll content, and Rubisco activity, enabling the genotype to maintain nutrient absorption and photosynthesis during the early period of waterlogging. However, compared to the control, both cowpea genotypes could not fully recover their photosynthetic capacity after 7 DOW, with a more significant decline in EpicSelect.4. Overall, our findings suggest that the tolerant UCR 369 genotype maintains higher photosynthesis under waterlogging stress attributable to higher photochemical efficiency, Rubisco activity, and less stomatal restriction. After recovery, the incomplete recovery of *A* can be attributed to the reduced g_s_ caused by severe waterlogging damage in both genotypes. Thus, promoting the rapid recovery of stomata from waterlogging stress may be crucial for the complete restoration of carbon fixation in cowpeas during the reproductive stage.

## 1. Introduction

Waterlogging stress limits crop yields in about 16% of global cultivated areas, and the problem is exacerbated in poorly drained soils [1,2]. For example, the wettest 12-month period in the United States, over 124 years of data, resulted in significant delays in crop planting dates as wet soil adversely affected yields [3,4]. Heavy precipitation events are projected to increase by about 7% for every 1 ºC increase in global warming, leading to increased flood hazard severity (high confidence) [5]. Furthermore, climate models predict a 20–40% increase in spring precipitation in the Mississippi River Alluvial Valley (MRAV) by the end of the 21st century [6]. The majority of cowpeas produced in the MRAV region are grown shortly after summer rains and then rely heavily on rainfall and irrigation for optimal growth and development [7]. Excessive precipitation can reduce yield by 10–90% in cowpeas, especially during the reproductive stage [8]. Therefore, it is crucial to understand how cowpea plants respond to waterlogging to reveal traits that contribute to tolerance.

Waterlogging first depletes oxygen levels in the soil by rapidly causing the diffusion rate of gases to drop by more than 10^4^ times relative to air [9]. Consequently, the soil redox potential of waterlogged soil decreases significantly [10], leading to hypoxia or anoxia [11], followed by reductions in essential soil elements, including NO_3_^−^, SO_2_^−^, Mn_4_^+^, and Fe_3_^+^ [12]. Under oxygen-deficient conditions, plant root metabolism changes from aerobic respiration to anaerobic fermentation, reducing plant energy by about 37.5% [13]. In addition, waterlogging can lead to an increase in CO_2_ concentrations in the plant root zone and a decrease in hydraulic conductance, resulting in the rapid closure of stomata [14]. Collectively, these changes in root functioning alter the energy resources of plants, preventing them from reaching their true genetic potential. Anaerobic conditions can adversely affect leaf physiology [2], nutrient absorption [15], enzymatic activity [16], plant growth and development [17], and ultimately lead to reduced crop yield and mortality. 

An important characteristic of plant responses to waterlogging is the alterations in shoot physiology, especially photosynthesis [2]. Previous studies have demonstrated the sensitivity of photosynthesis to waterlogging in cowpeas and related crops [7,18,19]. For instance, within the first day of waterlogging treatment, the carbon assimilation rate (*A*) of cowpea declined rapidly [7]. Thus, even in a short time, the significant reduction in *A* under waterlogging conditions could lead to a decline in plant energy reserves, indicating the existence of a common metabolic pattern. Imperatively, the factors affecting the *A* of plants are primarily divided into two distinct metabolisms: stomatal and non-stomatal limitations. Due to limited oxygen under waterlogging conditions, plants close their stomata to maintain plant water status, causing a decline in stomatal conductance (g_s_) and inhibiting the exchange of CO_2_ required by the plant’s basic processes [20]. Consequently, the reduction in g_s_ eventually leads to a corresponding decrease in *A* due to the decreased intercellular CO_2_ concentration (C_i_) under waterlogged conditions [21]. Another potential limitation of *A* in the submerged condition is the alteration in mesophyll conductance (g_m_), which is the diffusion of CO_2_ from intracellular space to the carboxylation site in the chloroplast stroma [22]. Non-stomatal limitation of *A* under waterlogging in legumes is associated with the maximum rate of Rubisco carboxylation (V_cmax_), ribulose-1,5-bisphosphate (RuBP) regeneration capacity mediated by maximum electron transport rate (J_max_), photosystem II (PSII) activity, Rubisco activity, and loss of pigments related to leaf senescence [14,23,24]. The effects of waterlogging on stomatal and non-stomatal factors limiting *A* varies with crop genotype, duration, and severity of waterlogging stress, ranging from a significant decline in sensitive genotypes to little or no inhibition in tolerant genotypes [2,25,26]. However, comparing these factors between waterlogging-tolerant and -sensitive genotypes is scarce in cowpeas. Hence, evaluating the key factors limiting the photosynthetic performance of cowpea genotypes could reveal the underlying mechanisms of their responses to waterlogging stress. 

Furthermore, decreased g_s_ in response to waterlogging may enhance the sensitivity of the photosynthetic apparatus to high irradiance, leading to photodamage of PSII due to overproduction of reactive oxygen species (ROS) [27]. The photodamage of photosystem II (PSII) affects the photosynthetic electron transport chain and alters the amount of light energy directed to organic synthesis, resulting in impaired chlorophyll fluorescence parameters [28]. Chlorophyll fluorescence is a rapid and non-invasive technique for screening diverse cultivars’ waterlogging tolerance [29]. Under waterlogged conditions, Ahmed et al. [30] found that the maximum quantum yield (F_v_/F_m_) of PSII, actual photochemical efficiency of PSII (Φ_PSII_), and photochemical quenching (qP) of mungbeans were downregulated, whereas the non-photochemical quenching (NPQ) was significantly increased. Conversely, for common bean cultivars with different waterlogging tolerances, F_v_/F_m_ was not affected by waterlogging, while the NPQ of susceptible cultivars increased with the duration of waterlogging treatments [31]. Thus, the sensitivity of legumes to waterlogging stress may be related to the decrease of PSII photochemistry and the enhancement of NPQ to dissipate excess energy.

Of the leguminous crops, cowpea is the most sensitive to waterlogging [32]. Most cowpea genotypes exhibited different responses to waterlogging stress depending on the growth stage to which the genotype was exposed [33]. Cowpea is tolerant during the vegetative stage [33], but becomes highly sensitive during early reproductive stages (R1–R3), resulting in a more than 50% reduction in seed yield [32]. Moreover, waterlogging tolerance at the reproductive stage is important in the MRAV regions of the United States, where high temperatures and relative humidity are expected to exacerbate the effects of late spring waterlogging stress. This is primarily because pollination and pod formation occurring at this stage directly contribute to economic yield. In addition, waterlogging events after flowering alter seed development by accelerating leaf senescence and nitrogen deficiency [34]. Nitrogen deficiency in cowpea leaves can be explained by the loss of chlorophyll pigment, which in turn slows the plant’s recovery after draining the waterlogged soil [35]. The ability of waterlogged plants to quickly restart normal physiological and metabolic activities after reoxygenation is critical for yield [13,36]. Thus, given recent climatic changes, there is an urgent need to exploit the natural variation in cowpeas for post-flowering waterlogging tolerance to develop more resilient, waterlogging-tolerant genotypes.

The objectives of this study were to investigate the relative responses of stomatal and non-stomatal factors affecting carbon fixation in cowpea genotypes during waterlogging and recovery period. We hypothesized that cowpea’s growth and physiological responses during and after waterlogging might differ with respect to waterlogging tolerance. This hypothesis was tested in two contrasting cowpea genotypes exposed to a 7-day waterlogging and recovery at the R2 stage using stomatal (g_s_ and transpiration rate; *E*) and non-stomatal (C_i_, g_m_, V_cmax_, J_max_, F_v_/F_m,_ chlorophyll content, etc.) factors. 

## 2. Results

### 2.1. Growth Responses of Cowpea Genotypes during and after Waterlogging

To evaluate the tolerance of cowpeas to waterlogging stress, two genotypes, ‘UCR 369’ and ‘EpicSelect.4’, were subjected to 7 days of waterlogging (DOW) and 7 days of recovery (DOR) at the R2 growth stages. Generally, 7 DOW caused a reduction in plant growth of both genotypes. Table 1 illustrates that waterlogging inhibited the shoot biomass accumulation of cowpeas despite the 7 DOR but in a genotype-dependent manner. At 7 DOW, waterlogged ‘UCR 369’ had a statistically comparable FM with the control treatment. However, the shoot FM of ‘EpicSelect.4’ differed significantly after 7 DOW by 37% compared to the control plants. At 7 DOR, the FM of waterlogged ‘UCR 369’ and ‘EpicSelect.4’ were significantly lower than the control plants (Table 1). Interestingly, the shoot DM of ‘EpicSelect.4’ declined faster than the ‘UCR 369’ when subjected to waterlogging. Specifically, waterlogged UCR 369 plants had significantly higher DM when compared to the control plants, whereas waterlogging significantly decreased the DM of ‘EpicSelect.4’ by 46% at 7 DOW (Table 1). At 3 and 7 DORs, waterlogged and control UCR 369 plants exhibited a statistically similar DM, while waterlogged EpicSelect.4 declined significantly by 56% and 47%, respectively, compared to the control plants (Table 1). In contrast, the DM/FM ratio of UCR 369 genotypes indicated significant increases of 13%, 14%, 21%, and 15% when subjected to 3 DOW, 7 DOW, 3 DOR, and 7 DOR, respectively. The DM/FM ratio for waterlogged EpicSelect.4 demonstrated similar trends only at 7 DOR but decreased significantly by 16% at 7 DOW.

RWC was measured to determine leaf water loss in cowpea genotypes during and after the waterlogging period. At 3 DOW, the mean RWC of waterlogged UCR 369 was statistically at par with the control (Table 1). However, after 7 DOW, the RWC of the waterlogged EpicSelect.4 decreased more rapidly (73% of the control plants) compared to the UCR, which decreased by less than 10%.

**Figure 1:**
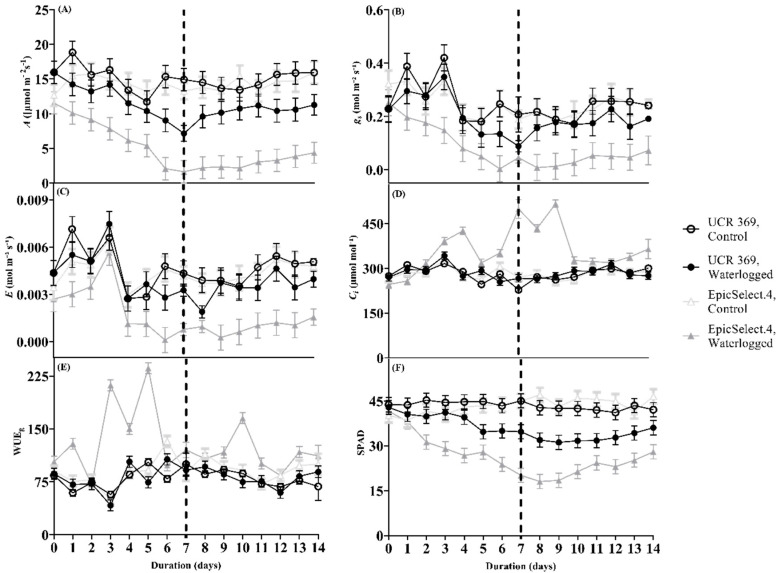
(**A**) CO_2_ assimilation rate (*A*), (**B**) Stomatal conductance (g_s_), (**C**) Leaf transpiration rate (E), (**D**) Intercellular CO_2_ concentration (C_i_), (**E**) Intrinsic water use efficiency (WUE), and (**F**) Chlorophyll content index (SPAD) of control and waterlogged cowpea genotypes (UCR 369 and EpicSelect.4) during and after 7 days of treatment. The dashed vertical lines in (**A**–**F**) demarcated the waterlogging from the recovery period. The error bar on the line graph indicates the standard error of the mean ± 5 replications of each leaf gas exchange trait. Standard error of the mean, *A* = 1.60; g_s_ = 0.049; E = 0.0.0008; C_i_ = 12.40; WUE = 7.93; SPAD = 2.39.

Moreover, waterlogged ‘EpicSelect.4’ showed a drastic reduction in SPAD values after 2 DOW, indicating early leaf senescence (Figure 1F), and most of the leaves dropped off by the end of the experiment. However, only slightly lower SPAD values for ‘UCR 369’ were evident after 1–4 DOW when compared to the control plants. The SPAD values of the two tested genotypes decreased gradually over time, with UCR 369 and EpicSelect.4 decreasing by 19% and 46%, respectively at 7 DOW compared to control. During the recovery, both cowpea genotypes could not restore leaf greenness with a consequent decline in SPAD values (Figure 1F).

### 2.2. Gas Exchange Responses of Cowpea Genotypes during and after Waterlogging 

Waterlogging treatments significantly affected the gas exchange parameters measured over the 7-day waterlogging and 7-day recovery period, depending on the genotype and duration of stress (Figure 1A–E, significant ‘genotype × treatment’ and ‘treatment × duration’, but not significant ‘genotype × duration’, and ‘genotype × treatment × duration’ interactions in Table 2). In UCR 369, waterlogging did not affect the stomatal conductance (g_s_) until 6 DOW, while the g_s_ of EpicSelect.4 significantly declined by 41% at 1 DOW (Figure 1B). Figure 1C shows the mirrored decrease in *E* over time for UCR 369 and EpicSelect.4 in control and waterlogged plants. Maintenance of *E* and g_s_ in UCR 369 compared to EpicSelect.4 suggests that water uptake continues due to open stomata until 6 DOW in UCR 369 (Figure 1B,C). In agreement, the *A* of UCR 369 was maintained up to 5 DOW but significantly decreased by 41% at the end of the waterlogging period (Figure 1A). This result contrasted with EpicSelect.4, where *A* significantly declined by 42% after 2 DOW and reached 2.07 µmol m^−2^ s^−1^ by 6 DOW compared to 14.30 µmol m^−2^ s^−1^ for control plants (Figure 1A). EpicSelect.4 did not restore g_s_, *E*, and *A* during the recovery, whereas the same parameters were statistically comparable to the control treatment in UCR 369 (Figure 1A–C).

The C_i_ of the waterlogged UCR 369 genotypes were not significantly different throughout the waterlogging period than the control plants (Figure 1D). However, waterlogging significantly increased the C_i_ of EpicSelect.4 from 271.57 µmol m^−2^ s^−1^ to 425.88 µmol m^−2^ s^−1^ at 4 DOW. A similar pattern of increased C_i_ was observed for EpicSelect.4 after 5 and 6 DOWs (Figure 1D). Measurements of WUE during waterlogging revealed a similar pattern in UCR 369, but there was a significant decrease in WUE of EpicSelect.4 after 4 to 6 DOWs followed by a drop during recovery (Figure 1E).

**Figure 2:**
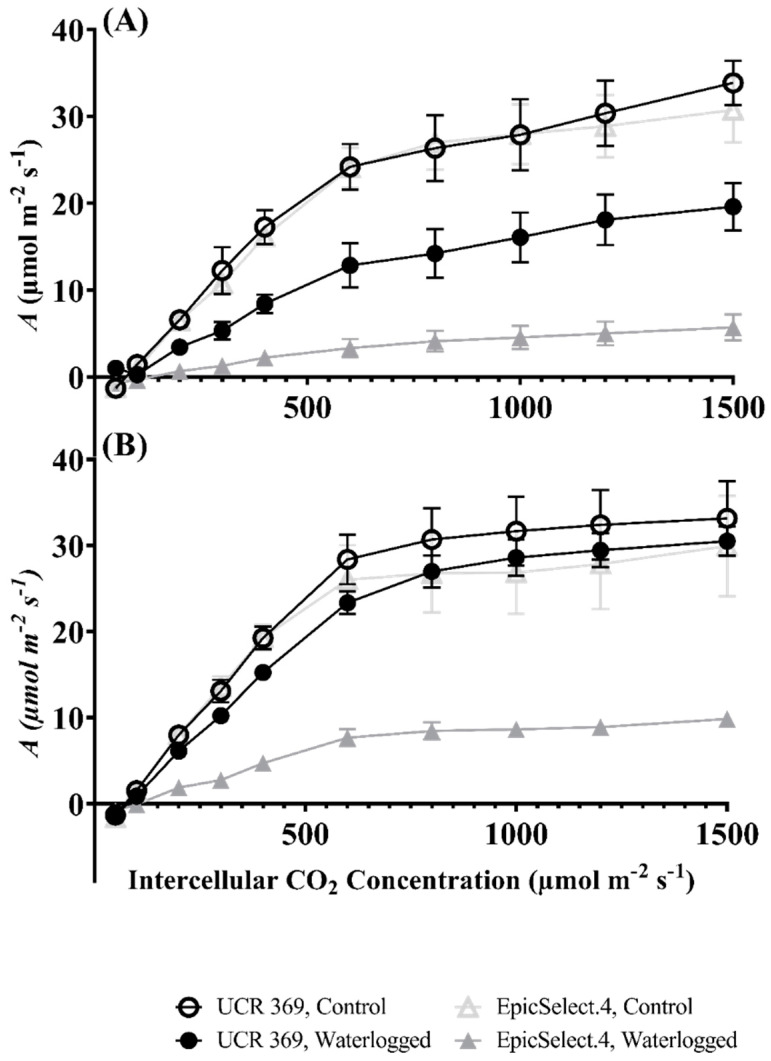
(**A**) Response of the CO_2_ assimilation rate (*A*) to increasing intercellular CO_2_ concentration (C_i_) (*A*/C_i_ Curve) in the two cowpea genotypes (UCR 369 and EpicSelect.4) after 7 days of control and waterlogging treatments; (**B**) *A*/C_i_ Curve in the two cowpea genotypes (UCR 369 and EpicSelect.4) after 7 days of recovery. The vertical bars represent the standard error of the mean (n = 5).

To investigate the biochemical limitation of *A*’s response in cowpeas under waterlogging, the *A*/C_i_ curve was measured at 7 DOW (Figure 2A) and 7 DOR (Figure 2B). The shape of the *A*/C_i_ curve varied between cowpea genotypes and waterlogging treatments (Figure 2). The *A* of the genotypes subjected to waterlogging and control treatments increased with increasing C_i_ from 0 to 1500 µmol mol^−1^ (Figure 2). Conversely, the *A* was lower under waterlogging compared to the control conditions, with a substantial decline in EpicSelect.4 compared to UCR 369 at 7 DOW (Figure 2A). After 7 DORS, the *A* of waterlogged UCR 369 was comparable to the control plants, while the waterlogged EpicSelect.4 could not restore their *A* compared to the control plants (Figure 2B). 

**Figure 3:**
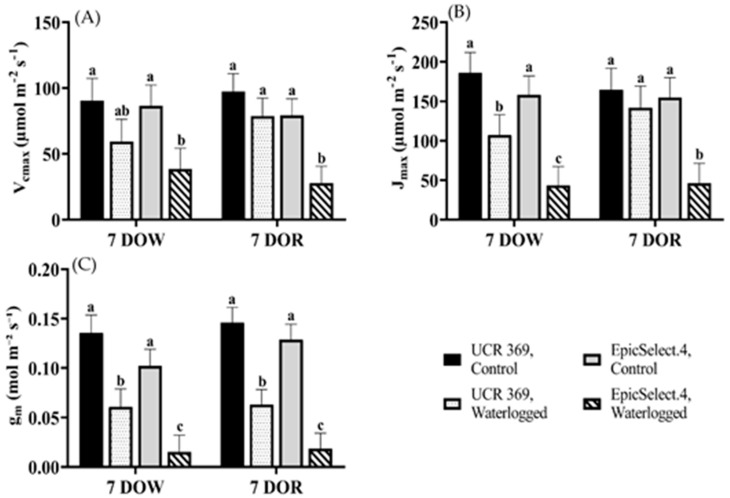
(**A**) Maximum rate of Rubisco carboxylation (V_cmax_), (**B**) Maximum rate of photosynthetic electron transport (J_max_), (**C**) Mesophyll conductance (g_m_) of control and waterlogged cowpea genotypes (UCR 369 and EpicSelect.4) after 7 days of waterlogging (DOW) and 7 days of recovery (DOR). Different lowercase letters indicate significant differences between genotype’s means and treatments (*p* < 0.05), as determined by Fisher’s LSD. The error bar on the vertical bar indicates the standard error of the mean ± 4 replications of each leaf gas exchange trait. Standard error of the mean at 7 DOW, V_cmax_ = 17.00; J_max_ = 25.80; g_m_ = 0.018; and at 7 DOR V_cmax_ = 13.70, J_max_ = 27.40, g_m_ = 0.015.

We used the *A*/C_i_ data of cowpea genotypes displayed in Figure 2 to estimate the mesophyll g_m_, V_cmax_, and J_max_ at both 7 DOW and 7 DOR. Waterlogging significantly affected the calculated V_cmax_ at 7 DOW. On average, UCR 369 had a significantly higher V_cmax_ of 90.22 µmol m^−2^ s^−1^ than EpicSelect.4 (86.52 µmol m^−2^ s^−1^) under control treatments (Figure 3A). Contrasted with nonwaterlogged plants, UCR 369 and EpicSelect.4 significantly reduced V_cmax_ by 31% and 48%, respectively, at 7 DOW. However, genotype and waterlogging treatment independently and significantly affected g_m_ and J_max_. Specifically, waterlogging significantly affected the g_m_ and J_max_ of UCR 369 by 55% and 34%, respectively, and by 85% and 73%, respectively, in EpicSelect.4 (Figure 3B,C). In addition, the g_m_, V_cmax_, and J_max_ declined in cowpea genotypes at 7 DORs, especially in EpicSelect.4, where g_m_, V_cmax_, and J_max_ significantly decreased by 83%, 65%, and 70%, respectively, in relation to the control plants. It is interesting to note that the values of g_m_, V_cmax_, and J_max_ of waterlogged UCR 369 after 7 DOR were statistically similar to the control plants (Figure 3). 

### 2.3. Photochemical Efficiency of Cowpea Genotypes during and after Waterlogging

**Figure 4:**
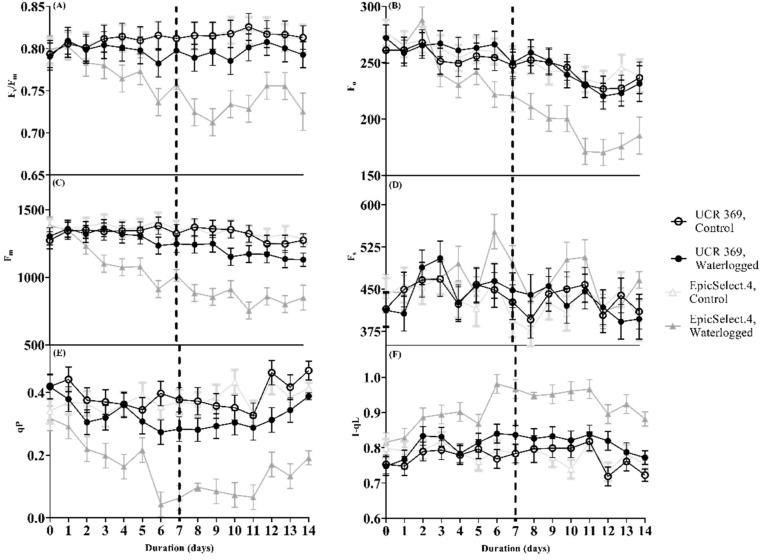
(**A**) Maximum quantum efficiency of PSII in dark-adapted state (F_v_/F_m_), (**B**) Initial fluorescence (F_o_), (**C**) maximum fluorescence (F_m_), (**D**) Steady-state fluorescence (F_s_), (**E**) Redox state of the plastoquinone pool (qP), and (**F**) Redox State of Plastoquinone Pool Based on the Lake Model (1-qL) of control and waterlogged cowpea genotypes (UCR 369 and EpicSelect.4) during and after 7 days of treatment. The dashed vertical lines in (**A**–**F**) demarcated the waterlogging from the recovery period. The error bar on the line graph indicates the standard error of the mean ± 5 replications of each leaf gas exchange trait. Standard error of the mean, F_v_/F_m_ = 0.16; F_o_ = 11.50; F_m_ = 63.30; F_s_ = 30.81; qP = 0.39; 1-qL = 0.027.

Analysis of chlorophyll fluorescence parameters (such as F_v_/F_m_, Φ_PSII_, and qP) is critical for evaluating the PSII photochemical efficiency in stressed plants [29]. For instance, F_v_/F_m_ reflects the PSII’s internal light energy conversion efficiency or maximum light [37]. The values of F_v_/F_m_ following 7 DOW showed the maximum quantum yield decreased over time but in a genotype-dependent manner (Figure 4; Table 2). Specifically, F_v_/F_m_ was observed to drop only at 6 DOW in UCR 369 relative to EpicSelect.4, where F_v_/F_m_ started declining by 3 DOW (Figure 4A), indicating the photoinhibition of PSII activity. In addition, the F_o_ and F_m_ of cowpea genotypes significantly decreased over time with the substantial decline in EpicSelect.4. Figure 4B reveals that the F_o_ values of UCR 369 under waterlogging were statistically comparable to the control plants. However, the F_o_ of EpicSelect.4 under waterlogging significantly decreased by 9% compared to the control plants after 3 DOW and continued declining throughout the experiment. A similar response was demonstrated in the F_m_ values, which showed a relatively stable trend in UCR 369 but a significantly decreasing trend in EpicSelect.4 from 3 DOW (Figure 4C). During the 1–7 DOR period, F_v_/F_m_, F_o_, and F_m_ values of UCR 369 showed statistically similar values to the control plants (Figure 4A–C). In contrast, the gradual reduction of F_v_/F_m_, F_o_, and F_m_ in EpicSelect.4 under waterlogging was an average of 10%, 22%, and 36%, respectively, of the control during the recovery period.

The redox state of qP, which measures the fraction of open PSII reaction centers, decreased significantly with the duration of waterlogging, compared to the control, but in a genotype-dependent manner (Figure 4E). The decline of qP in EpicSelect.4 was significant from 2 DOW, while in UCR 369, the decrease was statistically evident by 6 DOW. During 1–6 DOR, the qP of the waterlogged UCR 369 genotype was comparable to the control plants, whereas the fraction of closed PSII reaction centers was considerably increased in EpicSelect.4 (Figure 4E). Furthermore, the redox state of Q_A_ based on the lake model (1-qL) during 7 DOW and 7 DOR became more oxidized in waterlogged EpicSelect.4 from 2 DOW to the end of the experiment compared to the control plants (Figure 4F). Conversely, there was no significant difference between the 1-qL values of control and waterlogged UCR 369 throughout the experiment, except after 6 DOW and 5 DOR.

**Figure 5:**
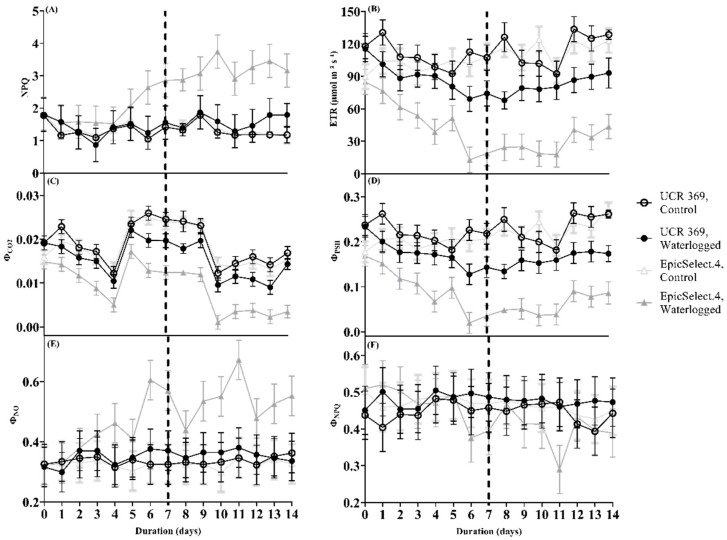
(**A**) Non-photochemical quenching (NPQ), (**B**) Electron transport rate (ETR), (**C**) Quantum yield of CO_2_ fixation (Φ_CO2_), (**D**) Effective quantum yield of PSII (Φ_PSII_), (**E**) Quantum yield of non-regulated energy dissipated in PSII (Φ_NO_), and (**F**) Quantum yield of regulated non-photochemical energy loss in PSII (Φ_NPQ_) of control and waterlogged cowpea genotypes (UCR 369 and EpicSelect.4) during and after 7 days of treatment. The dashed vertical lines in (**A**–**F**) demarcated the waterlogging from the recovery period. The error bar on the line graph indicates the standard error of the mean ± 5 replications of each leaf gas exchange trait. Standard error of the mean, NPQ = 0.51; ETR = 11.68; Φ_CO2_= 0.0016; Φ_PSII_ = 0.023; Φ_NO_ = 0.066; Φ_NPQ_ = 0.066.

When plants are subjected to environmental stress (such as water stress), they generate a pH gradient across the thylakoid resulting in the alterations of NPQ [38], which reflect heat dissipation using dark-adapted cowpea leaves. Subjecting UCR 369 to waterlogging did not induce substantial increases in NPQ throughout the experimental period (Figure 5A). However, in EpicSelect.4, by 6 DOW, NPQ significantly increased under waterlogging relative to the control plants. 

Moreover, we estimated the light energy partitioning at PSII on dark-adapted leaves, that is, Φ_PSII_, Φ_NPQ_, and Φ_NO_, which sum to one [39]. Compared to the control plants, waterlogging decreased the Φ_PSΙΙ_ of cowpeas, with the extent of decline dependent on cowpea genotypes and the duration of treatments (Table 2; Figure 5). In EpicSelect.4, which is the waterlogging-sensitive, the decrease in Φ_PSΙΙ_ was significant from 2 DOW, whereas in UCR 369, the more waterlogging-tolerant genotype lost its capacity to maintain Φ_PSΙΙ_ by 6 DOW and matched the drop in *A* (Figure 5D). It is interesting to note that a complete decline in Φ_PSΙΙ_ occurred throughout the recovery period with comparable values in waterlogged UCR 369 to control plants at 3 DOR. Corresponding responses were demonstrated for both cowpea genotypes during waterlogging and the recovery period with respect to the relative ETR of PSII reaction centers (Figure 5B), which is estimated as the multiplication of Φ_PSΙΙ_ by the irradiance level of the chamber. During waterlogging, the ETR decreased in parallel with the reduction in *A* for EpicSelect.4 and UCR 369 at 2 and 6 DOW, respectively (Figure 5B). 

Figure 5E shows no observable differences in the Φ_NO_ measured for control and waterlogged UCR 369 plants throughout the experiment. By contrast, waterlogged EpicSelect.4 showed significant differences in Φ_NO_ from 6 DOW to the end of the experiment in comparison with the control treatment (Figure 5E). Throughout the treatments, statistically similar patterns were observed in Φ_NPQ_ for waterlogged UCR 369 (Figure 5F). However, waterlogging significantly increased the Φ_NPQ_ of EpicSelect.4 at 1 DOW, remained comparable to the control between 2 and 5 DOW, and started declining from 6 DOW to 6 DOR (Figure 5F).

### 2.4. Relating Photochemical Efficiency in PSII with Leaf Gas Exchanges as Parameters for Waterlogging Tolerance

**Figure 6:**
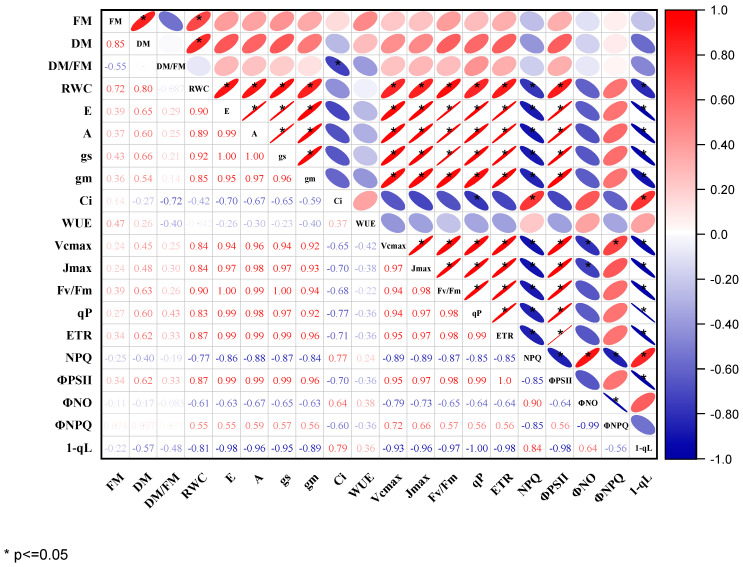
Pearson’s correlation matrix of the changes in biomass, gas exchange, and chlorophyll fluorescence parameters of the two cowpea genotypes under control and waterlogging treatments. Dark color represents strong correlations, and light background color represents weaker correlations. Values close to zero indicate no correlation, and values close to one indicate a strong correlation (positive—red and negative—blue) between two parameters. * Represent correlation coefficient significance levels at *p* ≤ 0.05. Where FM = fresh mass, DM = dry mass, RWC = relative water content, *E* = transpiration rate, *A* = CO_2_ assimilation rate, g_s_ = stomatal conductance, C_i_ = intercellular CO_2_ concentration, WUE = water use efficiency, V_cmax_ = maximum rate of Rubisco carboxylation efficiency, J_max_ = maximum rate of photosynthetic electron transport, F_v_/F_m_ = maximum quantum efficiency of PSII in dark-adapted state, qP = photochemical quenching, ETR = electron transport rate, NPQ = non-photochemical quenching, Φ_PSII_ = actual photochemical efficiency of PSII, Φ_NO_ = quantum yield of non-regulated energy dissipated in PSII, Φ_NPQ_ = quantum yield of regulated non-photochemical energy loss in PSII, 1-qL = redox State of Plastoquinone Pool based on the Lake Model.

Evaluated gas exchange and chlorophyll fluorescence parameters were highly correlated in Pearson’s correlation analysis (Figure 6). Except for C_i_, WUE, NPQ, Φ_NO_, and 1-qL, which showed a negative correlation, there was a significant and positive correlation between the biomass yields (FM; DM) of cowpeas with the gas exchange parameters. SPAD was positively correlated with most photosynthetic and chlorophyll fluorescence traits, indicating that increased stay-green leaf was associated with the higher photosynthetic performance of cowpea genotypes under waterlogging. Similarly, most photosynthetic traits (*A*, g_s_, *E*, g_m_, V_cmax_, J_max_) under waterlogging treatments were significantly and positively correlated with F_v_/F_m_, qP, ETR, Φ_CO2_, and Φ_PSII_, but negatively associated with NPQ, Φ_NO_, Φ_NPQ_, and 1-qL. However, the correlation coefficients of C_i_ with most parameters were in the range considered moderate to weak. Thus, suggesting C_i_ acts as a non-stomatal factor affecting the photosynthetic efficiency of cowpeas under waterlogging.

## 3. Discussion

Waterlogging is a type of abiotic stress that can affect plant growth and development [40]. Our previous study on screening 30 cowpea genotypes under waterlogging conditions classified 3 cowpea genotypes as tolerant and 20 as sensitive based on the waterlogging tolerance coefficient [7]. Understanding the limitations of photosynthesis in cowpea, a naturally waterlogging-sensitive plant, could elucidate the waterlogging tolerance mechanism of this species and improve cowpea breeding programs. In the present study, two genotypes of cowpeas UCR 369 (more waterlogging tolerant) and EpicSelect.4 (less waterlogging tolerant), were evaluated under 7 DOW duration and following 7 DOR. These contrasting cowpea genotypes were investigated through a comprehensive analysis of stomatal and non-stomatal factors affecting leaf carbon fixation and important growth traits.

Waterlogging significantly affects cowpeas’ shoot biomass but depends on growth stages and genotype. Relative to the response of cowpeas and related crops in previous studies, waterlogged-induced reductions recorded in the current study were lower. Most of these evaluations were done at the vegetative stage with over 50% biomass reduction [7,31,41]. In the current study, the biomass decline was less than half of the control in waterlogged genotypes at the R2 growth stages. After 7 DOW, EpicSelect.4 shoot biomass was reduced to 37–46% compared to the control, while waterlogged UCR 369 exhibited similar biomass to control and accumulated more shoot biomass at 3 DOW (Table 1). One explanation for the significant decline in the biomass of waterlogged EpicSelect.4 could be the accelerated leaf senescence and the limitation of nutrient absorption, as observed by the 46% decrease in chlorophyll content (SPAD value; Figure 1F) at 2 DOW. Waterlogging has previously been shown to have adverse effects on nutrient uptake in intolerant soybeans [42], cowpeas [32], common beans [43], and field peas [44]. Another reason for the biomass reductions could be the inability of the EpicSelect.4 to develop adventitious roots under waterlogging to compensate for the loss of damaged roots. Conversely, investment in shoot biomass in waterlogged UCR 369 is an interesting mechanism of waterlogging tolerance, presumably linked to the formation of adventitious roots and metabolites supplied to the shoot via the xylem, allowing them to be transported from the waterlogged root [19,45]. 

The ability of plants to maintain RWC under waterlogging and control plants have been widely used to understand waterlogging tolerance. At 7 DOW, there was a more significant decrease in the RWC of EpicSelect.4 compared to UCR 369, which retained more open stomata while maintaining leaf RWC. Analogous progressive decline in RWC under waterlogging has been reported in contrasting genotypes of pigeon peas [27,46]. Generally, sensitive plants such as EpicSelect.4 wilt on the first day of waterlogging due to reduced root hydraulic conductivity [47]. However, tolerant genotypes tend to rapidly open their stomata to better conserve water utilized for photosynthesis and transpiration [44]. This is another reason *A* was maintained in UCR 369 by sustaining *E* up to 5 DOW, while the *A* and *E* were drastically reduced in EpicSelect.4 under 2 DOW. It is also imperative to note that UCR 369 does not achieve waterlogging tolerance via water conservation. Instead, it was better able to maintain plant water status compared to EpicSelect.4 under waterlogging. This difference was more pronounced in the gas exchange response after 5 DOW (Figure 1), with EpicSelect.4 rapidly closing its stomata and reducing photosynthetic efficiency during and after waterlogging. 

Moreover, the capacity of plants to ensure comparable g_s_ with the control plants during 1–5 DOW may be responsible for the increase in the DM/FM ratio of waterlogged UCR 369 [48]. Rapid stomatal closure has been associated with decreased *A* in many plants [49], although it did not disrupt the *A* in the tolerant genotype under waterlogging in this study. Thus, waterlogged UCR 369 could continue accumulating metabolites and photoassimilates in plant tissues, leading to increased DM.

Although a contrasting mechanism of photosynthetic damage was demonstrated between tolerant UCR 369 and sensitive EpicSelect.4 genotypes, waterlogging significantly reduced *A* at 7 DOW for both cowpea genotypes (Figure 1). Previous studies have shown that stomatal closure is a key factor in lowering leaf carbon fixation in legumes due to limited CO_2_ supply to carboxylation sites under waterlogging stress. In the current study, A decline in UCR 369 was associated with a significant reduction of g_s_ (Figure 1A,B) without any biochemical reduction of V_cmax_ and J_max_ (Figure 3A,B). Thus, the photosynthetic downregulation in the tolerant genotype was primarily caused by stomatal-induced factors under waterlogging conditions. Ploschuk et al. [2] reported similar findings in wheat and barley, demonstrating tolerance under 2 weeks of waterlogging. In contrast, the sensitive EpicSelect.4 genotype experienced a significant decline in *A* with decreased g_s_, g_m_, V_cmax_, and J_max_ under waterlogging, indicating that both stomatal and non-stomatal limited photosynthesis according to the model of Farquhar et al. [50]. An increase in C_i_ relative to the control treatment was also observed in the EpicSelect.4 genotype during the progressive waterlogging (Figure 1D). Taken together, these findings suggest that the reduction in *A* in the sensitive genotypes was caused mainly by photosynthetic apparatus damage rather than a lack of intercellular CO_2_. Waterlogging intolerant genotypes such as rapeseed, field peas, and peanuts [2,51] have demonstrated *A* decreases, and C_i_ increases under waterlogging conditions. Islam et al. [52] also observed increased C_i_ in Vo1982A-G (sensitive mungbean genotypes) after 7 DOW and suggested that higher C_i_ limits Rubisco activity, resulting in plant inability to restore photosynthetic capacity during the recovery period. EpicSelect.4 also showed an increase in WUE under waterlogged conditions, suggesting that sensitive genotypes tended to gain less carbon per unit of water lost. An analogous pattern of increased WUE has been observed in waterlogging-sensitive legumes in previous studies [2,31,53]. Therefore, the photosynthetic capacity of EpicSelect.4 was far from recovery owing to a low CO_2_ intake compared to the tolerant UCR 369 genotype.

Many studies have evaluated the adverse effects of waterlogging on photosynthesis and how hypoxia and anoxia inhibit photosynthetic system activity by altering chlorophyll fluorescence parameters [29,54], thereby reducing leaf carbon fixation. Under waterlogging, the inactivation of PSII in field peas [2] and waxy corn [55] results in a loss in photosynthetic capacity with detrimental impacts on plant growth. Krause and Weis [56] opined that healthy leaves’ F_v_/F_m_ values vary from 0.75 to 0.83, and a reduction from these values indicates damaged PSII. In the current study, the F_v_/F_m_ of EpicSelect.4 genotype was significantly lowered at 3 DOW, and the decrease was below 0.75 from 6 DOW to the end of the experiment (Figure 4A). However, the F_v_/F_m_ of waterlogged UCR 369 ranged from 0.78 to 0.81 and was comparable to the control plants, ranging from 0.79 to 0.82. At the same time, compared with the control treatment, the F_o_ and F_m_ values of UCR 369 were statistically comparable, whereas these parameters were significantly decreased in EpicSelect.4 from 3 DOW and could not recover at 7 DOR (Figure 4B,C). The above results indicated that the PSII of the tolerant genotype remained stable under waterlogging conditions.

Conversely, waterlogging stress promoted PSII inhibition and stronger energy dissipation in sensitive cowpea genotypes. These results are consistent with the responses reported by Ploschuk et al. [2] in tolerant wheat and sensitive peas, as well as by Velasco et al. [31] in common beans. Colom and Vazzana [57] surmised that the reduction of F_v_/F_m_ under stress is associated with Φ_PSII_, resulting in increased thermal energy dissipation due to the inability of the xanthophyll cycle to protect *A* from photoinhibition in the PSII reaction center. Interestingly, we observed a positive correlation between Φ_PSII_ and F_v_/F_m_, demonstrating that the leaf F_v_/F_m_ can be used as an important index to evaluate the response of cowpeas to waterlogging stress.

Furthermore, waterlogging-induced *A* inhibition resulted in ETR downregulation in PSII. ETR decreased significantly at 2 DOW in EpicSelect.4 and 6 DOW in UCR 369 (Figure 5B), with data showing a high correlation with *A*. Throughout the experiment, similar patterns were displayed in the Φ_PSII_ of UCR 369 and EpicSelect.4. Since Φ_PSII_ is a measure of ETR in leaves, the higher value in UCR 369 implies that tolerant leaves have an improved capacity for converting light energy to chemical energy during photosynthesis [58]. Kramer et al. [39] reported that the energy absorbed in PSII is partitioned into three parts: Φ_PSII_, Φ_NPQ_, and Φ_NO_. The Φ_NPQ_ is a key indicator to measure the energy dissipation of light protection in plants, and the higher the Φ_NPQ_ value, the greater the capacity to remove excess light energy via enhanced heat dissipation systems [59]. The combined pathway of radiative and non-radiative deexcitation processes is represented as Φ_NO_, and increased Φ_NO_ means that the absorbed light energy cannot be fully utilized by photochemical energy conversion and photoprotective regulatory mechanisms [39,59]. In this study, waterlogged UCR 369 showed statistically similar values of Φ_NO_ and Φ_NPQ_ with the control plants (Figure 5E,F). In contrast, the waterlogged EpicSelect.4 increased Φ_NO_ and decreased Φ_NPQ_ at 6 DOW, indicating the sensitivity of its photosynthetic apparatus to non-regulated heat dissipation. Moreover, the 1-qL, which is the measure of the regulatory balance of the light reactions, became oxidized in EpicSelect.4 from 2 DOW (Figure 4F), indicating that a more significant proportion of the quanta in PSII were dissipated into heat and fluorescence. However, the stability of 1-qL waterlogged UCR 369 reveals that a larger percentage of the energy absorbed in PSII is converted to chemically fixed energy. Analogous to this study, previous research demonstrated that the Φ_PSII_, ETR, Φ_NPQ_ declined, and 1-qL and Φ_NO_ increased under waterlogging in sensitive peas [60], common beans [31], and sorghum [61]. Hence, lowering Φ_PSII_ and increasing 1-qL may lead to over-excitation of the photochemical system of EpicSelect.4 compared with UCR 369, which maintains leaf carbon fixation under waterlogging.

In addition to these parameters, Velasco et al. [31] reported that 14 DOW significantly decreased qP and increased NPQ in the sensitive common beans relative to tolerant genotypes. We found that progressive waterlogging significantly decreased qP and increased NPQ in EpicSelect.4 compared to no statistical change in either of these parameters in UCR 369. Zhang et al. [61] also demonstrated corresponding results for tolerant and sensitive sorghum genotypes under a 12-day waterlogging treatment. Tolerant UCR 369 maintained the proportion of open PSII reaction centers, thereby reducing heat dissipation under waterlogged conditions and enabling full utilization of light energy absorbed by leaves for photosynthesis and continued plant growth. Ma et al. [62] surmised that the capacity of waterlogging-tolerant genotypes to avoid photosynthetic apparatus damage when subjected to waterlogging is closely linked to improved chlorophyll and carotenoid content. Primarily, enhanced plant pigments promote the synthesis of numerous enzymes and electron transporters, resulting in better utilization of light energy received by the leaf in photochemical processes [59]. This resulted in the improved photosynthetic performance of UCR 369 under waterlogging, as shown by the Φ_PSII_, qP, and NPQ values. 

Overall, the results of this and earlier studies support the idea that improved chlorophyll fluorescence properties are a crucial factor influencing carbon fixation in the leaves of waterlogging-tolerant genotypes. Hence, the intrinsic processes of chlorophyll fluorescence properties relative to phytochromes (such as chlorophyll and carotenoids) under waterlogging conditions in contrasting cowpea genotypes can be further explored.

## 4. Materials and Methods

### 4.1. Plant Material and Growth Conditions

Two cowpea genotypes (EpicSelect.4 and UCR 369) with contrasting waterlogging tolerance as determined by Olorunwa et al. [7] were selected to investigate tolerance during and after waterlogging in the reproductive stage. The selected cowpea genotypes have similar growth and duration of the life cycle. This experiment was conducted in the Vegetable Physiology Greenhouse of the North Mississippi Research and Extension Center (Verona, MS) from 22 October to 21 December 2021. The greenhouse environment was set and recorded with a seed 16 controller (Wadsworth, Arvada, CO). Photosynthetic photon flux density (PPFD) inside the greenhouse was measured with an LI-190R quantum sensor (LI-COR, Inc., Lincoln, NE) connected to a CR1000x data logger (Campbell Scientific, Logan, UT). During the experiment, the average PPFD was 536.28 ± 11.7 μmol m^−2^ s^−1^. Plants were held at a temperature of 30/20 °C (day/night) for a 16/8 h period, respectively. Also, the average relative humidity during the experiment was 63%, 64%, and 70%, respectively, for October, November, and December 2021. 

The cowpea seeds were inoculated before sowing with *Bradyrhzobium japonicum* (Visjon Biologics, Wichita Falls, TX) at the rate of 141 g per 22.68 kg of seeds. Four inoculated cowpea seeds of each genotype were planted into one-gallon pots filled with Pro-Mix BX soilless medium (Premier Tech Horticulture, Quebec, Canada) and watered daily. Twice a week, the plants were fertigated with a 5-15-29 water-soluble nutrient solution at the rate of 100 ppm. Plants were thinned to one plant per pot at 14 days after sowing (DAS). After 45 DAS, cowpea plants were subjected to two experimental treatments consisting of waterlogging and control treatments at the R2 growth stage.

### 4.2. Waterlogging Treatments

Cowpea plants were waterlogged by placing 6 pots of each cowpea genotype into five replicated containers (Husky 15-gallon Latch and Stack Tote, Home Depot, Atlanta, GA). To simulate 7 DOW treatments, the container was filled with tap water to a height of 2–3 cm above the substrate surface. Pots containing cowpea plants were maintained at optimal field capacity under the control treatments. After 7 DOW, the pots were removed from the 15-gallon container filled with water, and plants were allowed to recover for additional 7 days. 

### 4.3. Gas Exchange Measurements

Parameters related to gas exchange were measured on the second, most fully expanded trifoliate at 1 to 7 DOW and 1 to 7 days of recovery (DOR). The *A*, g_s_, C_i_, and *E* were measured in situ with chlorophyll fluorescence at the North Mississippi Research and Extension Center (10.00–14:00 CST) using an LI-6800 portable photosynthesis system (LI-COR, Biosciences, Lincoln, NE, USA). Measurements were allowed to match the chamber environment before the values were recorded. The chamber environment was set to match the growth chamber, with 1500 µmol m^−2^ s^−1^ of light intensity, 415 ppm of CO_2_ concentration in the air (C_a_), and 50% relative humidity. Measurements were conducted on five representative plants of each cowpea genotype subjected to waterlogging and non-waterlogging treatments during waterlogging and recovery. The ratio of *A*/g_s_ was used to calculate intrinsic water use efficiency (WUE) [63].

Additionally, the CO_2_ response curves (*A*/C_i_) measurements were evaluated using the auto program settings in the LI-6800 at 7 DOR and 7 DOR. To measure the steady-state response of *A*/C_i_, the leaf chamber settings were fixed at 50% relative humidity, 1500 µmol m^−2^ s^−1^ light intensity, and the temperature set to maintain ambient greenhouse temperature (28–30 °C). Using the built-in program on the LI-6800, measurements were taken at 50, 100, 200, 300, 400, 500, 600, 800, 1000, 1200, and 1500 ppm CO_2_, with early matching enabled and wait times of 60–90 seconds between measurements. *A*/C_i_ analyses were performed according to Sharkey et al. [64], with few changes as portrayed in Olorunwa et al. [65] using the excel fitting tool 10.0 available at http://landflux.org/Tools.php. Representative individual curves were fitted separately, and the extracted parameters were averaged across replicates for each treatment. According to Bernacchi et al. [66], the estimated *A*/C_i_ response curve was further utilized to calculate the maximum rate of Rubisco carboxylation (V_cmax_), the maximum rate of photosynthetic electron transport (J_max_), and mesophyll conductance (g_m_).

### 4.4. Chlorophyll Fluorescence Measurements

The LI-6800 using pulse-amplitude modulated (PAM) fluorometry with a Multiphase Flash Fluorometer (6800-01A, LI-COR Biosciences, Lincoln, NE, USA) was used to measure the chlorophyll fluorescence at 1 to 7 DOW and 1 to 7 DOR. During predawn hours (3:00–5:00 CST), the minimal fluorescence (F_o_) was measured on the second-most fully expanded leaf using a measuring light (0.005 µmol m^−2^ s^−1^). The maximal fluorescence (F_m_) was quantified using a 1-second saturating pulse at 8000 µmol m^−2^ s^−1^ in dark-adapted leaves. The leaves were continuously illuminated for 20 min with an actinic light (1400 µmol m^−2^ s^−1^) to record the steady-state yield of fluorescence (F_s_). Maximal light-adapted fluorescence yield (F′_m_) was determined by 8000 µmol m^−2^ s^−1^. The actinic light was turned off, and minimal fluorescence yield (F′_o_) in the light-adapted state was determined after 5 s of far-red illumination. The difference between the measured values of F_m_ and F_o_ is the variable fluorescence (F_v_). The chlorophyll fluorescence parameters were calculated using the following formulas [67,68].

F_v_/F_m_ = (F_m_ − F_o_)/F_m_ (1)

Φ_PSII_ = (F′_m_ − F_s_)/F′_m_ (2)

Φ_NPQ_ = F_s_/F′_m_ − F_s_/F_m_ (3)

Φ_NO_ = F_s_/F_m_ (4)

qP = (F′_m_ − F_s_)/(F′_m_ − F′_o_) (5)

NPQ = F_m_ − F′_m_/F′_m_ (6)

where F_v_/F_m_ is the maximal photochemical efficiency of PSII, Φ_PSII_ is the actual photochemical efficiency of PSII, Φ_NPQ_ is the quantum yield for the energy dissipated via Δ pH and xanthophyll-regulated processes, Φ_NO_ is the quantum yield of non-regulated energy dissipated in PSII, and qP and NPQ are the photochemical and the non-photochemical quenching, respectively. The electron transport rate (ETR) of chlorophyll fluorescence was calculated according to Genty et al. [67].

### 4.5. Plant Growth, Relative Water Content, and Harvest

The chlorophyll content index (CCI) of the functional leaves was measured at 1 to 7 DOR and 1 to 7 DOR using a SPAD (soil and plant analysis development) analyzer (SPAD-502 Chlorophyll Meter, Konica Minolta, Tokyo, Japan). The relative CCI of each leaf, represented by the SPAD value, can be used to study the effect of waterlogging on leaf yellowing in cowpea genotypes associated with nitrogen remobilization and leaf senescence. Three readings were collected from each cowpea genotype’s top-most fully expanded trifoliate and averaged.

Five representative cowpea plants (from each treatment/replications/genotype) were harvested on 3 DOW, 7 DOW, 3 DOR, and 7 DOR to obtain growth data on the effects of waterlogging stress. The plant component, fresh mass (FM), was measured using a weighing scale from all plants. Plant FM samples were lyophilized using a FreeZone 2.5 L freeze dryer (Labconco Corp., Kansas City, MO, USA) to determine the dry mass (DM) and percent dry mass (%DM). The cowpea’s relative water content (RWC) was determined as per the method of Barrs and Weatherley [69] with minor modifications. The RWC value is estimated as ((FM − DM/TM − DM) × 100). TM is the turgid mass, determined by soaking the FM of five replicated plants per treatment per genotype in distilled water and then obtaining the weight after 24 h.

### 4.6. Statistical Analysis

The experiment was a randomized complete block design with two waterlogging treatments, two cowpea genotypes, five replications, and twelve plants in a factorial arrangement. In total, 240 plants (5 replicates × 2 waterlogging treatments × 2 cowpea genotypes × 12 plants) were utilized in this study. SAS (version 9.4; SAS Institute, Cary, NC, USA) was used to perform a statistical analysis of data. A three-way analysis of variance (ANOVA) using the generalized linear mixed model (PROC GLIMMIX) was used to assess the effects of factors (treatments, genotypes, and duration), along with their interactions, on the replicated values of CCI, gas exchange, and chlorophyll fluorescence parameters. The experiment’s fixed effects consist of treatment, genotypes, and duration, where the replication (5 levels) was treated as a random effect. The responses of FM, DM, and RWC values were analyzed by a two-way ANOVA with ′genotype’ and ′treatment’ as the main factors. Fisher’s protected least significant difference tests *p* ≤ 0.05 were employed to test the differences between the interactions of factors for measured parameters. The standard errors of the mean were calculated using the pooled error term from the ANOVA table and presented in the figures as error bars. Diagnostic tests, such as Shapiro–Wilk in SAS, were conducted to ensure that treatment variances were statistically equal before pooling. A Pearson correlation analysis was utilized to study the relationship between the studied parameters. Graphs were plotted with GraphPad Prism 9 (version. 9.1.0; GraphPad Software Inc., San Diego, CA, USA).

## 5. Conclusions

In this study, gas exchange and chlorophyll fluorescence parameters were evaluated to reveal the key factors influencing leaf carbon fixation and the adaptive mechanism of cowpea genotypes under waterlogging stress. After 7 DOW and 7 DOR, the tolerant UCR 369 genotype exhibited superior plant growth and photosynthetic efficiency than the waterlogged sensitive genotype, EpicSelect.4. This study confirmed that the ability of UCR 369 to develop adventitious roots and maintain biomass accumulation are critical for waterlogging tolerance. Moreover, the analysis of gas exchange traits revealed that the photosynthetic response to waterlogging differed between tolerant and sensitive cowpea genotypes. At 6 DOW, the downregulation of *A* was mainly driven by decreased g_s_ and g_m_, with no biochemically limiting declines in V_cmax_ and J_max_, as well as chlorophyll fluorescence parameters. However, the sensitive EpicSelect.4 showed a significant decrease in *A* at 2 DOW, with a corresponding reduction in g_s_, g_m_, V_cmax_, J_max_, F_v_/F_m_, qP, ETR, and Φ_PSII_ under waterlogged conditions, indicating that stomatal and non-stomatal limited photosynthesis is taking place when the genotype is waterlogged stressed. These waterlogging-induced photosynthetic changes are consistent with rapid leaf chlorosis in cowpea genotypes based on the SPAD values and chlorophyll fluorescence data. 

Moreover, the downregulation of Φ_PSII_, Φ_NPQ_, and qP values in PSII at 2 DOW indicated that sensitive EpicSelect.4 could not absorb energy for photochemical reactions, resulting in damaged photosynthetic apparatus. At the same time, the elevated values of NPQ, 1-qL, and Φ_NO_ in EpicSelect.4 compared to UCR 369 may partly contribute to photoinhibition and decreased photochemical efficiency during waterlogging. Further studies evaluating carotenoid and chlorophyll content are needed to understand the light-dependent response mechanism in tolerant and sensitive cowpea genotypes.

## Figures and Tables

**Table 1:** Fresh (FM), dry mass (DM), dry mass to fresh mass ratio (DM/FM %), and relative water content (RWC) of UCR 369 and EpicSelect.4 cowpea genotypes under control and waterlogging treatments during 3 days of waterlogging (harvest 1), 7 days of waterlogging (harvest 2), 3 days of recovery (harvest 3), and 7 days of recovery (harvest 4).

		FM (g/plant)	DM (g/plant)	DM/FM (%)	RWC (%)
		**UCR 369**			
**HARVEST 1** **(3 DOW)**	Control	92.68 ± 4.63 b	16.59 ± 1.16 h	17.79 ± 0.56 b	83.30 ± 1.13 f
	Waterlogging	89.60 ± 5.42 b	17.93 ± 0.92 gh	20.50 ± 0.36 a	84.27 ± 3.18 ef
		**EpicSelect.4**			
	Control	148.45 ± 6.33 a	24.07 ± 1.22 bc	16.20 ± 0.39 c	96.19 ± 0.60 a
	Waterlogging	137.66 ± 7.31 a	21.84 ± 1.24 cde	15.86 ± 0.24 c	80.09 ± 1.60 g
**HARVEST 2** **(7 DOW)**	**UCR 369**				
	Control	119.22 ± 5.17 b	21.28 ± 0.81 def	18.07 ± 0.72 b	86.55 ± 0.46 cd
	Waterlogging	120.02 ± 4.21 b	24.43 ± 0.55 bc	20.22 ± 0.35 a	77.91 ± 0.84 gh
	**EpicSelect.4**				
	Control	170.07 ± 5.90 a	24.63 ± 1.19 b	14.40 ± 0.34 c	93.83 ± 1.33 b
	Waterlogging	107.60 ± 10.60 c	13.42 ± 1.55 i	12.10 ± 0.42 d	68.95 ± 1.76 i
**HARVEST 3** **(3 DOR)**	**UCR 369**				
	Control	135.42 ± 8.10 b	22.43± 1.30 bcd	16.33 ± 0.33 b	87.62 ± 0.92 cd
	Waterlogging	98.82 ± 3.76 c	19.40 ± 0.58 fg	19.76 ± 0.32 a	78.29 ± 0.99 g
	**EpicSelect.4**				
	Control	176.74 ± 8.27 a	22.94 ± 1.10 bcd	12.98 ± 0.09 c	96.06 ± 0.80 a
	Waterlogging	76.94 ± 2.53 d	10.12 ± 0.44 j	13.16 ± 0.42 c	79.40 ± 0.35 g
**HARVEST 4** **(7 DOR)**	**UCR 369**				
	Control	119.39 ± 7.10 b	21.85 ± 1.14 cde	18.43 ± 0.31 b	85.96 ± 1.04 cde
	Waterlogging	93.65 ± 3.04 c	19.75 ± 0.64 ef	21.12 ± 0.32 a	81.57 ± 1.05 fg
	**EpicSelect.4**				
	Control	182.83 ± 3.22 a	27.79 ± 0.64 a	15.21 ± 0.28 d	96.47 ± 0.57 a
	Waterlogging	115.82 ± 5.58 b	19.86 ± 0.69 efg	17.39 ± 0.40 c	76.09 ± 0.73 h
Treatment		***	**	***	***
Genotype		***	NS	***	NS
Harvest		***	***	***	***
Treatment * Genotype		***	**	NS	***
Treatment * Harvest		***	***	NS	*
Genotype * Harvest		***	***	***	***
Treatment * Genotype * Harvest		NS	*	NS	***

Note: Different low case letters across a column indicate interaction effects at *p* ≤ 0.05 by Fisher’s least significant difference. The values are means ± standard errors of 5 replicates. *, **, and *** represent statistical significance at *p* ≤ 0.05, 0.01, and 0.001, respectively. NS represents no statistically significant *p* > 0.05.

**Table 2:** F-values of three-way ANOVA (factors: ‘genotype, ‘treatment’, and ‘duration) for gas exchange and chlorophyll fluorescence parameters of UCR 369 and EpicSelect.4 cowpea genotypes under 7 days of waterlogging and 7 days of recovery treatments.

Studied Parameters	Source of Variation						
	Genotype (G)	Treatment (T)	Duration (D)	G × T	G × D	T × D	G × T × D
CCI (SPAD)	111.76 ***	878.91 ***	10.36 ***	108.32 ***	1.82 *	14.56 ***	3.07 ***
*A* (μmol m^−2^ s^−1^)	110.44 ***	335.44 ***	9.06 ***	72.78 ***	0.67 ^NS^	4.53 ***	1.39 ^NS^
g_s_ (mol m^−2^ s^−1^)	51.08 ***	66.43 ***	14.06 ***	34.51 ***	0.59 ^NS^	3.04 **	0.72 ^NS^
*E*(mol m^−2^ s^−1^)	66.64 ***	94.43 ***	17.9 ***	45.59 ***	0.72 ^NS^	3.22 ***	0.85 ^NS^
C_i_(μmol m^−1^ )	7.40 **	11.83 **	2.87 **	8.49 **	2.22 *	2.11 *	2.26 *
WUE	16.13 ***	8.00 **	1.32 ^NS^	8.21 **	0.89 ^NS^	0.88^NS^	1.69 ^NS^
F_v_/F_m_	40.14 ***	120.79 ***	1.43 ^NS^	40.34 ***	1.48 ^NS^	3.85 ***	1.34 ^NS^
F_m_	69.74 ***	273.48 ***	13.44 ***	111.26 ***	3.44 ***	8.19 ***	2.20 *
F_o_	28.44 ***	43.42 ***	26.82 ***	58.71 ***	2.97 ***	3.58 ***	2.44 **
F_s_	4.06 *	3.85 *	4.03 ***	5.54 *	1.3 ^NS^	1.32^NS^	1.07 ^NS^
F′_m_	6.99 **	36.04 ***	3.24 ***	0.64 ^NS^	1.09 ^NS^	2.16 *	0.76 ^NS^
F′_o_	21.29 ***	19.4 ***	4.65 ***	13.08 ***	1.68 ^NS^	1.17 ^NS^	0.90 ^NS^
F′_v_/F′_m_	0.56 ^NS^	37.77 ***	3.92 ***	0.42^NS^	1.7 ^NS^	2.18 *	0.97 ^NS^
Φ_PSII_	108.87 ***	316.93 ***	6.19 ***	68.53 ***	0.78 ^NS^	5.49 ***	2.23 *
Φ_CO2_	109.72 ***	332.55 ***	68.34 ***	71.87 ***	0.66 ^NS^	4.45 ***	1.38^NS^
ETR	108.87 ***	316.93 ***	6.19 ***	68.53 ***	0.78 ^NS^	5.49 ***	2.23 *
NPQ	5.33 *	7.33 **	1.45 ^NS^	4.36 *	0.8 ^NS^	1.06 ^NS^	0.82 ^NS^
qP	114.35 ***	285.72 ***	7.28 ***	84.05 ***	1.19 ^NS^	5.15 ***	2.65 **
qN	2.34 ^NS^	31.39 ***	16.08 ***	0.06	1.38 ^NS^	2.33 **	0.95 ^NS^
1-qL	86.12 ***	205.19 ***	7.05 ***	69.8 ***	1.16 ^NS^	4.34 ***	2.09 *
Φ_NO_	7.41 **	9.36 **	1.01 ^NS^	7.87 **	0.83 ^NS^	1.00 ^NS^	0.84 ^NS^
Φ_NPQ_	5.52 *	5.91 *	0.94 ^NS^	6.3 *	0.82 ^NS^	0.88 ^NS^	0.77 ^NS^

Note: *, **, and *** represent statistical significance at *p* ≤ 0.05, 0.01, and 0.001, respectively. NS represents not statistically significant *p* > 0.05. Where CCI = chlorophyll content index, *A* = CO_2_ assimilation rate, g_s_ = stomatal conductance, *E* = transpiration rate, C_i_ = intercellular CO_2_ concentration, WUE = water use efficiency, F_v_/F_m_ = maximum quantum efficiency of PSII in dark-adapted state, F_m_ = maximum fluorescence, dark-adapted, F_o_ = initial fluorescence, dark-adapted, F_s_ = steady-state fluorescence, F′_m_ = maximum fluorescence, dark-adapted, F′_o_ = initial fluorescence, dark adapted, F′_v_/F′_m_ = maximum quantum efficiency of PSII in the light-adapted state, Φ_PSII_ = actual photochemical efficiency of PSII, Φ_CO2_ = quantum yield of CO_2_ fixation, ETR = electron transport rate, NPQ = non-photochemical quenching, qP = photochemical quenching, qN = non-photochemical quenching, 1-qL = redox State of Plastoquinone Pool based on the Lake Model, and Φ_NPQ_ = quantum yield of regulated non-photochemical energy loss in PSII.

## Data Availability

The data supporting this study’s findings are available from the corresponding author upon reasonable request.
